# Impact of postpartum metritis on the regeneration of endometrial glands in dairy cows

**DOI:** 10.3168/jdsc.2022-0338

**Published:** 2023-07-21

**Authors:** I. Sellmer Ramos, J.G.N. Moraes, M.O. Caldeira, S.E. Poock, T.E. Spencer, M.C. Lucy

**Affiliations:** a Division of Animal Sciences, University of Missouri, Columbia, MO 65211; b Department of Animal and Food Sciences, Oklahoma State University, Stillwater, OK 74075; c College of Veterinary Medicine, University of Missouri, Columbia, MO 65211

## Abstract

The postpartum uterus involutes to its pre-pregnant and fully functional state within approximately 60 d after calving. Uterine glands are essential for fertility but little is known about their regeneration postpartum. Likewise, the effect of uterine disease (metritis) on gland regeneration is unknown. We hypothesized that uterine glands would be regenerated early postpartum and that metritis would be associated with slower gland regeneration to affect their numbers later postpartum during the breeding period. Postpartum dairy cows were diagnosed as healthy (n = 17 and 9 for experiment [Exp.] 1 and 2) or metritis (n = 17 and 10 for Exp. 1 and 2, respectively) at 7 to 10 d postpartum. Cows were slaughtered at approximately 1 mo (Exp. 1) or approximately 80 or 165 d (Exp. 2) postpartum for the collection of the uterus. Uterine tissue was sectioned and the number of glandular cross-sections per unit area was counted and cross-sectional area measured. Cellular proliferation within the luminal epithelium (LE) and glandular epithelium (GE) was quantified by MKI67 (marker of cellular proliferation) immunohistochemistry. In early postpartum cows (Exp. 1), the greatest amount of MKI67 staining was found in the deep endometrium (cells closest to the myometrium). Cows with purulent material in the uterine lumen at d 30 slaughter (Exp. 1) had fewer endometrial glands per unit area in the deep and middle endometrium when compared with nonpurulent cows. The MKI67 staining was less in the deep endometrial GE and LE for purulent compared with nonpurulent cows. Estrus cyclicity was associated with a greater number of gland cross-sections in the deep and middle endometrium. Later postpartum (80 and 165 d; Exp. 2), there was greater glandular development compared with Exp. 1 and a tendency for a lesser number of gland cross-sections per unit area in diseased cows without an effect on MKI67 staining in the GE or LE. We conclude that uterine disease slows the development of uterine glands early postpartum (by 1 mo) through a mechanism that involves cellular proliferation within the GE. The impact of the early postpartum disease on glandular development later postpartum (Exp. 2) appeared to be less. Additional time, therefore, may allow recovery of the GE in later postpartum cows.

## Highlights

• Glandular epithelial cell proliferation was greater in the deep endometrium of the postpartum uterus.
• Metritis was associated with less glandular development and less epithelial proliferation within the deep endometrium.
• Estrus cyclicity was associated with a greater number of gland cross-sections in the endometrium.
• Later postpartum cows had greater glandular development when compared with early postpartum cows.
• Cows with metritis tended to have less glandular development in the deep endometrium around breeding time.

## Graphical Abstract

**Figure:**
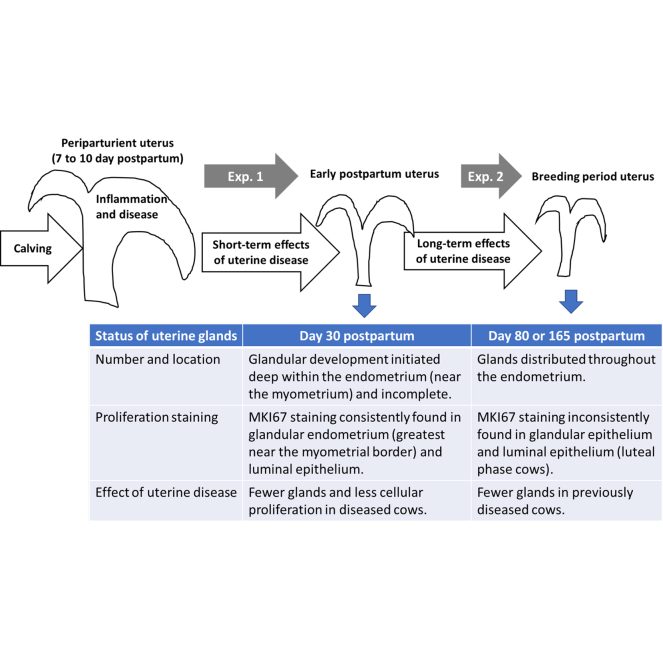
**Summary:** We examined the possibility that metritis is associated with reduced uterine glandular development as a mechanism to explain lesser fertility in cows with early postpartum uterine disease. The development of uterine glands early postpartum was greater in the deep endometrial layer (cells closest to the myometrium). Cows with metritis had less glandular development and less cellular proliferation within the glandular epithelium at 1 month postpartum. Compared with early (1 month) postpartum, there was greater glandular development in later postpartum cows (during the breeding period) and a tendency for an effect of metritis on the extent of glandular development (reduced in metritic cows). We concluded that postpartum metritis reduces the development of glands during the first month postpartum and this effect may be maintained into the breeding period.

Uterine involution is defined by a series of immunological and morphological events that return the uterus to its pre-pregnant state. During uterine involution, the luminal epithelium (**LE**) grows across the surface of the denuded caruncle. Reestablishment of the LE postpartum creates a physical barrier between the lumen of the uterus and the endometrial stroma of the cow. Approximately 10 to 25% of cows will develop uterine disease (metritis) after calving (Sheldon et al., 2020). Pathogenic bacteria gain direct access to the underlying stroma through sloughed LE and also by destroying the LE (cytolysis) to create a chronic inflammatory state (Bromfield et al., 2015; Sheldon et al., 2020). Abnormal uterine physiology caused by chronic inflammation may be an underlying cause of long-term infertility in dairy cows that experience uterine disease early postpartum (LeBlanc et al., 2002).

Although the speed and completeness of LE reestablishment during uterine involution is clearly important, less is known about the regeneration of endometrial glands in the postpartum cow. Given the importance of endometrial glands and the glandular epithelium (**GE**) to bovine fertility (Spencer et al., 2019), we designed 2 studies that addressed gland regeneration and the possible impacts of uterine disease postpartum in the short and long term. The objectives were to examine uterine glandular development and cellular proliferation in healthy and metritic cows at approximately 30 d postpartum (**dpp**; experiment [**Exp.**] 1) and later postpartum (approximately 80 and 165 dpp; Exp. 2). Our hypothesis was that metritis early postpartum (diagnosed 7 to 10 dpp) or the presence of purulent material in the uterine lumen at 30 dpp (Exp. 1) would be associated with slower development of glands through an effect on cellular proliferation. Later postpartum (80 and 165 dpp), we hypothesized that metritis early would be associated with reduced total number of glands. Data from postpartum cows (Exp. 1 and 2) were compared with nulliparous heifers (positive control for nongravid, nondiseased uterus).

Study procedures were approved by the University of Missouri (**MU**) Institutional Animal Care and Use Committee (protocol number 9635). For Exp. 1 and 2, first parity Holstein cows were selected from a confinement herd in eastern Kansas or the MU herd. Cows with a single clinical diagnosis of metritis at 7 to 10 dpp (fetid red-brown watery vaginal discharge with a flaccid uterus) were selected and matched with clinically healthy postpartum cows (viscous [not watery] and nonfetid discharge; single clinical diagnosis) that calved during the same week. Evidence that supports the clinical diagnosis made on-farm includes greater plasma haptoglobin in metritic versus healthy cows at the time of diagnosis for Exp. 1 (2.84 ± 0.40 vs. 0.35 ± 0.40 g/L; *P* < 0.001) and Exp. 2 (1.21 ± 0.24 vs. 0.22 ± 0.28 g/L; *P* < 0.020; metritis vs. healthy, respectively; ELISA assay; Immunology Consultants). According to the data of Huzzey et al. (2009) haptoglobin above 1 g/L and below 0.5 g/L within 1 wk postpartum would indicate metritis and healthy cows, respectively. There were also greater 16S rRNA read counts for typical metritis pathogens (e.g., *Fusobacterium necrophorum*, *Porphyromonas levii*, and *Bacteroides*) in uterine swabs collected at clinical diagnosis from metritis versus healthy cows (Exp. 1, Lucy et al., 2022; Exp. 2, M. C. Lucy and J. C. C. Silva, University of Missouri, unpublished).

For Exp. 1, there were 17 metritis and 17 healthy cows. Metritic and healthy cows were randomly assigned to either antibiotic treatment (healthy; n = 9 and metritis, n = 8; ceftiofur hydrochloride [i.m.; 2.2 mg/kg for 3 d]) or not treated (healthy, n = 8 and metritis, n = 9). Kansas herd cows in Exp. 1 were moved to MU shortly after diagnosis. Experiment 1 cows were slaughtered at approximately 30 dpp (29.1 ± 1.7 dpp; mean ± SD). Based on analyses of thrice-weekly plasma progesterone before slaughter using a validated RIA, there were 3 out of 17 metritic cows and 7 out of 17 healthy cows cycling (>1 ng/mL progesterone) before slaughter.

For Exp. 2, all cows were selected from the Kansas herd used in Exp. 1 but stayed in the Kansas herd until 1 d before slaughter at approximately 80 dpp (5 metritis and 5 control; 79.0 ± 7.5 dpp; mean ± SD) or approximately 165 dpp (5 metritis and 4 control; 165.0 ± 4.9 dpp; mean ± SD). Preliminary results from Exp. 1 demonstrated no long-term effect of antibiotic treatment on a variety of study endpoints including microbiome and inflammation (Lucy et al., 2022). Cows in Exp. 2, therefore, were treated with ceftiofur hydrochloride at the discretion of the herdsman. Cows in Exp. 2, were treated with a sequence of 2 PGF_2α_ injections at a 14-d interval before slaughter to target the luteal phase at slaughter. Blood samples were collected every other week for cows in Exp. 2. Based on plasma progesterone >1 ng/mL before and at the time of slaughter, all cows in Exp. 2 were cycling with an average interval to cyclicity of 36.2 ± 14.3 dpp (mean ± SD).

Holstein heifers (n = 10) from the MU herd (approximately 1 yr of age; slaughtered at d 14 of the estrous cycle) were used as a never-pregnant nondiseased control for comparison with Exp. 1 and 2 cows. Cows or heifers were slaughtered by captive bolt and exsanguination at the MU abattoir. The reproductive tract was removed, placed on ice in a plastic bag, and transported to the laboratory. The uterine lumen was flushed with sterile saline. The saline flush was classified visually as either clear (nonpurulent, no pus) or containing purulent material (pus). A cross-section from each uterine horn that included both caruncular and intercaruncular areas was then fixed in 10% neutral buffered formalin.

The fixed tissues were paraffin embedded and subjected to hematoxylin and eosin staining and immunohistochemistry for MKI67 (cellular proliferation marker) and FOXA2 (gland specific cell marker). The primary antibodies were against MKI67 (clone RM360; 1:500 dilution; Bio SB) or against FOXA2 (ab108422; 1:500 dilution; Abcam). Subsequent reactions were performed using reagents supplied with the Vectastain Elite ABC-HRP Kit (PK-6101; Vector Laboratories). Sections were counterstained with Harris hematoxylin stain.

Histological sections were subjected to morphological evaluation using ImageJ (National Institutes of Health). The endometrium in cross-section was divided into thirds defined as deep (region closest to the myometrium; stratum basalis), middle (region half-way between the myometrium and LE; stratum spongiosum), and superficial (region closest to the uterine lumen; stratum compactum; Figure 1A). Photographs were captured from each region (deep, middle, and superficial) for the previously gravid and nongravid horns (1 and 2 photographs per horn, respectively; Exp. 1) or ipsi- and contralateral horns to the corpus luteum (1 and 2 photographs per horn, respectively; Exp. 2) from each individual cow. A 200× magnification was used for the photographs (Leica DM4000 B microscope fitted with a Leica DFC 450C camera; Leica Microsystems). The number of uterine gland cross-sections per microscopic field (0.33 mm^2^) was counted and the area of each gland cross-section was measured by tracing around the basement membrane of the epithelium and calculating an area using the measurement tool in ImageJ (Figure 1B). “Gland area” refers to the average of the cross-sectional area of the glands that were counted in a field. Data were averaged before analysis when duplicate photographs were analyzed. The labeling index (percentage of MKI67 positive GE or LE cells) was determined by counting a minimum of 100 GE or LE cells and calculating the percentage with positive staining (brown color) for MKI67 (Figure 1B, 1C, and 1D).

**Figure 1:**
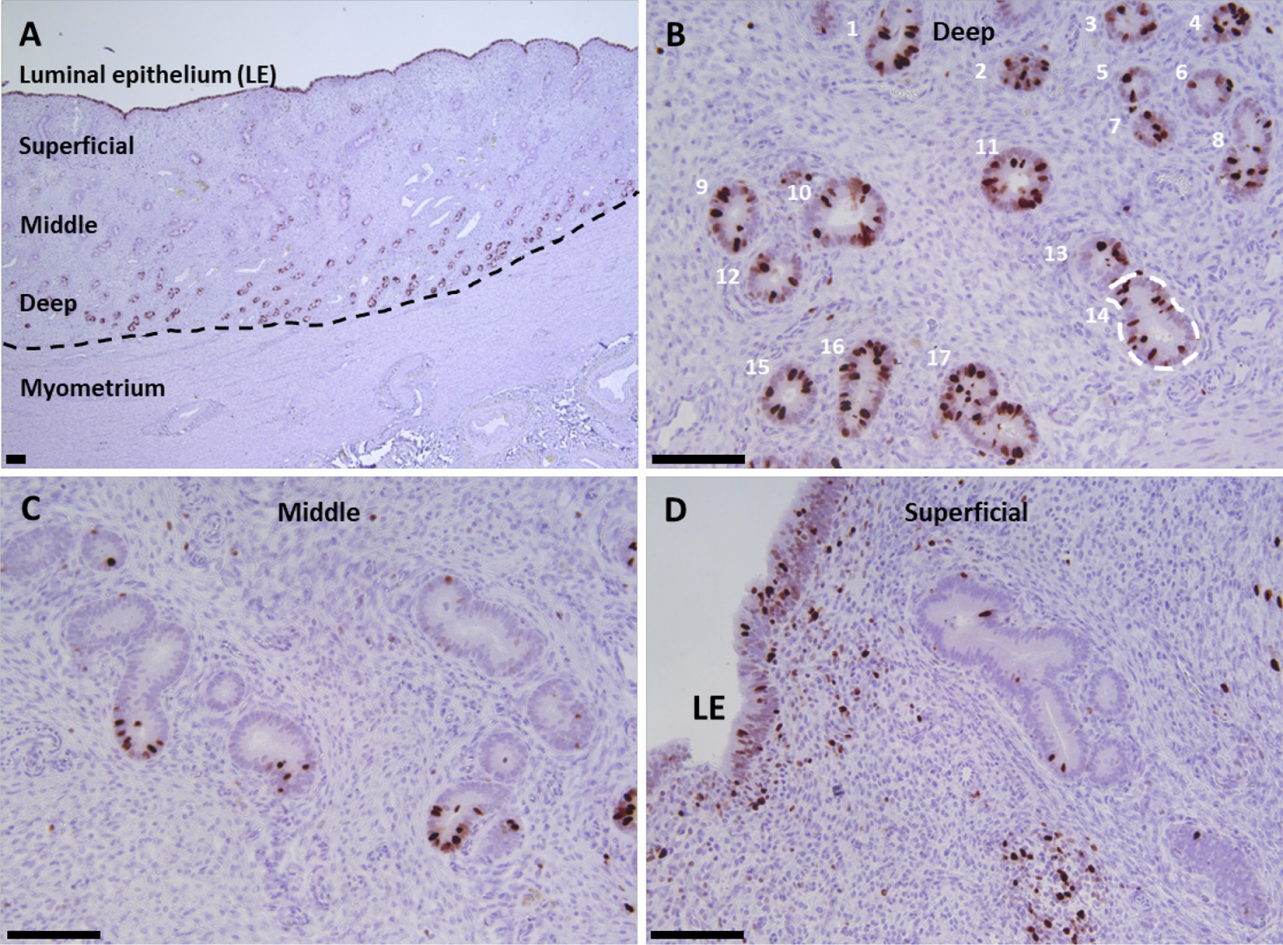
System for counting number of gland cross-sections, gland area, and MKI67 (cellular proliferation marker) labeling index. Endometrial cross-sections were divided into thirds defined as deep, middle, and superficial (A). The number of uterine gland cross-sections per microscopic field (0.33 mm^2^) was counted (B; 17 cross-sections) and the area of each gland cross-section was measured by tracing around the basement membrane of the epithelium (B; dashed line within cross-section 14). The MKI67 labeling index was determined by counting a minimum of 100 glandular epithelium (GE) or luminal epithelium (LE) cells and calculating the percentage with positive staining (brown color) for MKI67 (B, deep; C, middle; and D, superficial). Bar = 100 µm.

The dependent variables were (1) the number of gland cross-sections per microscopic field; (2) the average area (µm^2^) of glandular cross-sections; and (3) the MKI67 labeling index. All data were analyzed using PROC MIXED of SAS 9.4 (SAS Institute Inc.). A full model that included status (healthy or metritic), treatment (antibiotic or control), layer (deep, middle, or superficial), horn (previously gravid/nongravid or ipsi/contralateral to the corpus luteum), cyclicity (based on plasma progesterone before slaughter), and all interactions was initially fit. The effect of antibiotic treatment was not significant (*P* > 0.10), so a reduced model (status, layer, horn, cyclicity, status by layer, status by horn, and so on) was tested. A second reduced model that included the effects of flush phenotype at slaughter (clear or purulent; Exp. 1 only) was also tested. For Exp. 2, all cows were cycling before slaughter and all flushes were clear so the effects of cyclicity and flush phenotype were not tested. In a final set of analyses, Exp. 1 cows, Exp. 2 cows, and heifers were compared for the number of gland cross-sections and gland cross-section area. Least squares means were separated by using the PDIFF procedure. Significance was declared at *P* < 0.05. A statistical tendency was 0.05 < *P* < 0.10. Data are presented as least squares means ± standard error of the mean unless stated otherwise.

In Exp. 1, there was an effect of location (*P* < 0.001) on the number of gland cross-sections, the gland cross-section area, and MKI67 index (Table 1). The greatest number of gland cross-sections was found in the deep (10.4 ± 0.4) compared with middle (7.1 ± 0.4) or superficial (3.4 ± 0.4) endometrium (*P* < 0.001; Table 1; Figure 1). Conversely, gland cross-section area (µm^2^) was least in the deep layer (7,051 ± 1,252) and increased in the middle (10,072 ± 1,252) and superficial endometrium (18,102 ± 1,252; *P* < 0.001; Table 1; Figure 1). Based on MKI67, cells within the GE and LE were proliferating (Figures 1, 2A, and 2B). Gland cross-sections in the deep endometrium had a MKI67 index (17.9 ± 1.9) that was nearly twice what was observed for middle (7.1 ± 1.9) or superficial (10.3 ± 1.9) endometrium (*P* < 0.001; Table 1; Figure 1). The MKI67 index for the LE (15.3 ± 1.9) was similar to the deep endometrium (Figure 1). Positive MKI67 staining was observed in all cows (100%) in the deep endometrium, 29 cows (85.3%) in the middle endometrium, and 32 cows (94.1%) in the superficial endometrium and 33 cows (97.1%) in the LE.

**Table 1:** Least squares means and SE for the number of gland cross-sections per 0.33 mm^2^ field, the area of the glands (μm^2^), and MKI67 labeling index for deep, middle (Mid), and superficial (Sup) endometrium in primiparous Holstein cows that were diagnosed as healthy or having metritis from 7 to 10 d postpartum with tissue collected at 30 d postpartum (experiment 1) or 80 and 165 d postpartum (experiment 2)^1^

Item	Original diagnosis (7 to 10 d postpartum)								*P*-value^2^		
	Healthy (n = 17)				Metritis (n = 17)						
	Gland location				Gland location						
	Deep	Mid	Sup	SE	Deep	Mid	Sup	SE	Status	Loc	S × Loc
Experiment 1											
Gland no.	11.2	7.1	3.0	0.5	9.6†	7.1	3.9	0.7	0.725	0.001	0.028
Gland area	8,186	11,169	21,621	1,533	5,918	8,975	14,584**	1,979	0.057	0.001	0.135
MKI67 index	21.4	9.4	11.6	2.4	14.4†	4.8	8.9	3.0	0.095	0.001	0.658
Final status (30 d postpartum, uterine flush)											
	Clear (n = 20)				Purulent (n = 14)						
Gland no.	11.3	7.6	3.5	0.5	8.4**	5.8*	2.8	0.8	0.012	0.001	0.119
Gland area	7,350	10,499	18,767	1,483	6,753	9,930	18,552	2,481	0.839	0.001	0.991
MKI67 index	21.7	7.2	11.3	2.1	9.6**	7.1	12.7	2.5	0.258	0.002	0.007
	80 and 165 d postpartum (combined)										
Experiment 2	Healthy (n = 9)				Metritis (n = 10)						
Gland no.	29.4	16.9	6.5	1.6	25.6†	16.6	7.3	1.5	0.549	0.001	0.118
Gland area	2,388	4,836	12,243	1,526	2,953	5,271	15,317	1,476	0.354	0.001	0.545
MKI67 index	15.2	4.9	1.7	5.1	9.3	5.5	4.8	4.9	0.910	0.006	0.246

1 Cows in experiment 1 were also classified as having clear or purulent uterine flush at slaughter.

2 Loc = location; S × Loc = interaction of status × location. Differences between least squares means for healthy versus metritis within gland location are indicated as **P < 0.01, *P < 0.05, and †*P* < 0.10.

**Figure 2:**
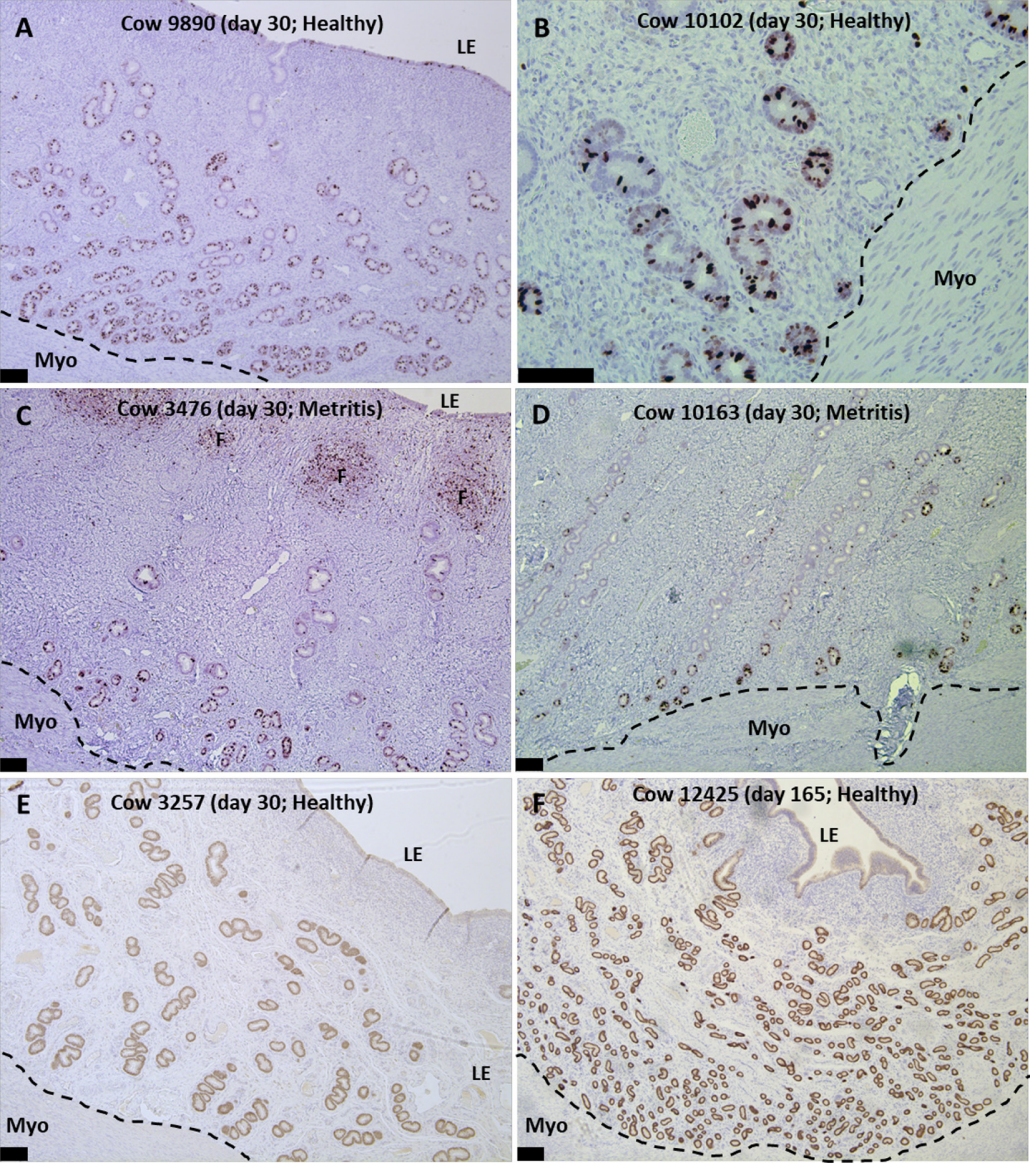
Immunohistochemical sections of healthy cows at 30 d postpartum (dpp; A and B; experiment 1; MKI67 stain; MKI67 was used to specifically identify proliferating cells), metritis cows at 30 dpp (C and D; experiment 1; MKI67 marker stain), a healthy cow at 30 dpp (E; experiment 1; FOXA2 stain; FOXA2 used to specifically label uterine glands), and a healthy cow at 165 dpp (F; experiment 2; FOXA2). Myo = myometrium; LE = luminal epithelium; F = lymphocytic foci; bar = 100 µm.

Cows in Exp. 1 diagnosed with metritis had fewer gland cross-sections in the deep endometrium (Table 1; status by location; *P* < 0.028; Figure 2A, 2B, 2C, and 2D). Compared with clear flush, cows with purulent flush in Exp. 1 had fewer gland cross-sections in the deep (*P* < 0.002) and middle (*P* < 0.047) endometrium (status; *P* < 0.012). Fewer gland cross-sections in the deep endometrium of purulent cows were associated with a reduction in MKI67 index specifically in the deep endometrium (status by layer; *P* < 0.007; Table 1; Figure 2A, 2B, 2C, and 2D). The MKI67 index in the LE was also reduced for cows with purulent (8.6 ± 3.7) compared with clear (17.9 ± 2.2) uterine flush (*P* < 0.037).

There was an interaction of cyclicity and location (*P* < 0.006) for number of gland cross-sections because cyclic cows had more gland cross-sections in the deep (12.2 ± 0.8 and 8.0 ± 0.5; *P* < 0.001) and middle (8.1 ± 0.8 and 6.0 ± 0.5; *P* < 0.029) endometrium when compared with noncyclic cows (respectively). We did not detect an effect of cyclicity (*P* > 0.10) on MKI67 index in the GE.

For Exp. 2, there was an effect of dpp (80 vs. 165) on the number of gland cross-sections (fewer glands at d 165; 19.4 ± 1.2 vs. 14.7 ± 1.3; *P* < 0.010) and area of gland cross-section (larger area at d 165 specifically in the superficial location; 9,895 ± 1,442 vs. 17,665 ± 1,526; location by day *P* < 0.009). The effect of horn was not significant. Later postpartum cows (Exp. 2) were similar to early postpartum cows (Exp. 1) for the effects of location on number of glands and gland area (decrease in the number of gland cross-sections with an increase in area from deep to superficial endometrium; Table 1). Cows diagnosed with metritis tended to have fewer gland cross-sections compared with healthy cows, but this effect was restricted to the deep glands (*P* < 0.092; Table 1). Compared with early postpartum (Exp. 1), MKI67 staining was less consistent for the Exp. 2 cows. There were 11 cows (57.9%) positive for MKI67 within deep endometrium, 7 cows (36.8%) positive in middle endometrium, 5 cows (26.3%) positive in superficial endometrium, and 6 cows (31.6%) positive in the LE. There was an effect of location on the MKI67 index (greatest in the deep endometrium; Table 1) but no effect of the initial disease diagnosis (healthy vs. metritis; Table 1).

Data from the Exp. 1 and 2 postpartum cows were compared with the nulliparous heifers. The number of gland cross-sections were greatest in deep endometrium in heifers (54.9 ± 1.5) compared with Exp. 2 (27.4 ± 1.1) or Exp. 1 (9.3 ± 0.8) cows (*P* < 0.001). For the number of middle gland cross-sections, heifers (20.5 ± 1.5) and Exp. 2 cows (16.9 ± 1.1) were similar but greater than Exp. 1 cows (6.6 ± 0.8; *P* < 0.001). The number of superficial gland cross-sections were least in Exp. 1 cows (*P* < 0.01; 3.3 ± 0.8, 6.9 ± 1.1, and 6.3 ± 1.5 for Exp. 1, Exp. 2, and heifers, respectively).

We found a greater number of gland cross-sections in the deep compared with middle or superficial endometrium (effect of location; Table 1; Figure 1). We also observed, particularly in early postpartum cows, that the deep endometrium had GE with the greatest MKI67 index (Figure 1A, 1B, 2A, and 2B). The bovine morphology resembles closely the morphology of glands described in the human where endometrial glands deep in the endometrium are found in a mesh- or mycelium-like network (Yamaguchi et al., 2022). Glands reaching the uterine lumen project perpendicularly toward the surface (Figure 2D).

The number of gland cross-sections increased from early postpartum (Exp. 1) to later postpartum (Exp. 2; Figure 2E and 2F). There was also nearly twice the MKI67 index for deep glands compared with middle or superficial glands in Exp. 1 (Figures 1, 2A, and 2B). We assume that GE proliferation represents gland regeneration early postpartum. If true, the early postpartum cow is different from newborn animals where new glands arise from the LE and develop downward toward the myometrium (Spencer et al., 2019).

Gland formation began early postpartum and continued until at least 80 dpp. The MKI67 index for later postpartum cows (Exp. 2) was less and also less consistent when compared with early postpartum cows (Exp. 1) where nearly all glands were labeled. Uterine cell proliferation was shown to be dependent on the stage of the estrous cycle (Arai et al., 2013). Early postpartum (Exp. 1), we detected an effect of cyclicity on the number of gland cross-sections in the deep and middle endometrium (greater number in cyclic cows). Estrous cycles via changes in progesterone or estradiol may promote endometrial development (Arai et al., 2013) and these same mechanisms may stimulate glandular development early postpartum.

The endometrium of parous cows (Exp. 1 and 2) had fewer gland cross-sections compared with nulliparous heifers. One speculation is that the greater density of glands in heifers compared with cows explains better overall fertility in heifers (Dhaliwal et al., 2002). This intriguing possibility will require further study with the caveat that there are many differences between the reproductive biology of cows and heifers.

There were fewer gland cross-sections in the deep and middle endometrium for cows with early postpartum uterine disease (Exp. 1; Table 1). This was associated with a lower MKI67 index in diseased cows (Table 1). Based on our subjective histological observations, it appeared that glands that were developing early postpartum did not penetrate the subepithelial stroma if the subepithelial stroma was highly inflamed (Figure 2C).

We observed that the number of gland cross-sections in the deep endometrium tended to be reduced in the later postpartum cows with early postpartum uterine disease (Exp. 2; Table 1). Although there was not a substantial reduction in gland number, gland function may have been compromised in diseased cows. Whether the number or function of uterine glands can explain, in part, the long-term effects of early postpartum uterine disease on the fertility of dairy cattle is an open question arising from this work.

In conclusion, 2 important questions that are relevant to bovine fertility postpartum were answered by this study. First, we described the quantity and distribution of glands in early postpartum cows and identified the deep endometrium as an active area of cellular proliferation within the GE. It appeared that glands regenerate from within the deep endometrium early postpartum (Exp. 1). Later postpartum (d 80 and 165; Exp. 2), there were more gland cross-sections within the deep endometrium compared with d 30 (Exp. 1), but the later postpartum cows did not achieve the same density of glands that was found in nulliparous heifers. Second, uterine disease slows the development of glands early postpartum (Exp. 1) and may reduce the total number of glands in the deep endometrium later postpartum (Exp. 2). The impact of glandular development on fertility of the postpartum cow and whether a reduction in gland density or function explains lesser fertility in cows with early postpartum uterine disease are 2 additional questions arising from this research that will require further study.
